# Medical and Philosophical Intelligence

**Published:** 1817-07

**Authors:** 


### MEDICAL AND PHILOSOPHICAL INTELLIGENCE.

ROYAL SOCIETY.—On Thursday, May 1, a paper, by Sir EVERARD HOME, on the Passage of the Ovum from the Ovarium into the Uterus, was read. Very little light has hitherto been thrown on this subject. Hervey, though supplied with deer by royal munificence, failed in his investigations. John Hunter was equally unsuccessful with sheep. Accident threw into the author's way an observation which serves to cast a new light upon this obscure subject. A female servant, aged 23, was absent from home about four hours, and returned in high spirits. She fell ill in the evening, had an epileptic fit and fever, and died in a week. On examining the body after death, the uterus gave signs of having been impregnated. She had been impregnated a week before death. The ovum was in the uterus, enveloped in coagulated lymph; but Mr. Bower was able, by his skill in using the microscope, to examine it, and to determine its nature unequivocally. It had come from the ovarium on the left side, which was of a larger size than the other. Two corpora lutea were observable, and there were several cavities from which ova had previously made their escape. Sir Everard conceives that these ova make their escape occasionally, whenever any great excitement of the system takes place. The semen of the male makes its way to the uterus, and the impregnation takes place there. He conceives that the ovum remains in contact with the male semen for several days to complete the impregnation.—*Annals of Philos.*

We have chosen to copy the above, not having been present when the paper was read. We shall defer our remarks till it appears in the "Transactions."

On the following Thursday, a paper was read, by Sir Everard, on an improved Method of preparing the Colchicum Autumnale for medicinal use. We have often been accused of prematurely offering the minutes of learned Societies before the publication of their Transactions. We acknowledge some inconveniences occur from it; and, if we could always ascertain which papers would be printed, we should be careful never to notice them till they appear regularly before the public. The last-mentioned paper being a continuation of one in the former volume of the Philosophical Transactions, we shall, in our next, notice the two together, and also what other physiological papers the volume may contain.

On the 15th May, a valuable paper was read from that accurate experimenter Dr. DAVY, who, to his honour, employed so usefully the leisure of a long voyage. "The general result of these observations is, that the specific gravity of the sea is nearly the same every where. He does not agree with a modern traveller of high authority, who considers the specific gravity of sea-water to differ in every zone. The small differences that exist are not easily accounted for. In one case he found the specific gravity diminished after very heavy rain, rain. It was generally altered by squally weather. In general, the temperature of the air was highest exactly at noon, and lowest just at sun-rise; but in a perfect calm the temperature of the air was the same as on land, namely, its greatest height was some time after noon. The reason is, that heat accumulates both in the ship and in the sea."

---

At the sittings of the *Faculty of Medicine* at Paris, on the 12th of June, 1817, M. Chaussier presented the singular case of a woman who had borne *eleven* children; and who, on dissection, was found to want the left ovarium entirely. A memoir is preparing on the subject.

On the same day, Baron Larrey presented a young gentleman who, in fencing, broke his adversary's foil, which (the plunge being made at his face,) entered the head at the left nostril, and even touched the brain. His faculties are deranged; and, though he always recognised the Baron, he had forgotten his name, and, indeed, cannot recollect the name of scarcely any thing. The right side is paralysed, and the *left* side of the tongue, while the right side of it enjoys healthy action. The Baron intends to read a memoir accounting for the singularities presented in this case, which we shall give.

Professor Thillaye, who had been charged by the Faculty with a report on Dr. Adams's Life of the late John Hunter, read it to the Society, at the same time. M. Thillaye executed his task with all that care which the learned body of which he is a member, and the author's reputation, demanded. He paid a just eulogium to Dr. Adams on his important rectifications of errors that had gone abroad, and gave an extensive analysis of the volume, translating the more interesting passages. We must observe that a *written* report on a work presented to the Faculty is a rare honour, and the highest compliment to the author of a work, their reports being ordinarily *verbal*; and, what adds still more to that honour is, that the illustrious Professor Chaussier should propose its being printed in the Bulletin of the Faculty.

We have before expressed our regret that so few honours await the profession of medicine, on which account we are always thankful when we receive such notices as the above. To this we may add the distinguished manner in which our valued correspondent Mr. Stevenson has received an unsolicited appointment:—

"In consideration of the personal benefits received from the professional talents of John Stevenson, Esq. of Great Russel-street, Bloomsbury-square, his Royal Highness the Duke of York has been graciously pleased to appoint him his Surgeon-Oculist and Aurist."

The distinction paid to Dr. Davis is not less creditable to the public than flattering to himself.

"No cases can possibly occur to a practitioner of midwifery combining more difficulty and distress than those in which it is necessary to destroy the child in order to preserve the life of the mother;

 and and any facility that can be afforded to such operation, when circumstances demand its performance, must be regarded as a benefit both to the profession and to society, of no inconsiderable moment. We were, therefore, much gratified to hear that the gold medal of the first class was presented by the Society of Arts, on the day of the annual distribution of premiums (27th of May last), to Dr. Davis, of Charlotte-street, Bloomsbury, for the only substantial improvement in this department of the profession which has been made for upwards of half a century, i. e. since the time of Smellie."

A description and drawing of this instrument will be given in our next.

---

The anniversary meeting of the *Philosophical Society of London* was held at the Society's Rooms, adjoining Scot's Corporation Hall, Crane-court, Fleet-street, on Thursday the 12th of June. The following noblemen and gentlemen were chosen officers and council for the ensuing year:

*President*—Right Hon. the Earl of Carysfort, K.P. F.R.S. F.A.S. D.C.L.

*Vice-Presidents*—Right Hon. Lord Henniker, F.R.S. F.A.S.; Sir J. C. Hippisley, Bart. M.P. LL.D. F.R.S. F.A.S.; Isaac Hawkins Browne, F.R.S.; Rev. W. B. Collyer, D.D. F.A.S.; Olinthus Gregory, LL.D.; Rev. Abraham Rees, D.D. F.R.S. F.L S.; James Sowerby, F.L.S. G.S. W.S.; J. F. Vandercom, F.G.S.

*Treasurer and Honorary Secretary*—Thomas Joseph Pettigrew, F.L.S.

*Registrar*—John Miers.

*Assistant ditto*—T. K. Cromwell.

*Curators*—W. C. Pettigrew and T. J. Armiger.

*Orator for* 1818—John Mason Good, F.R.S.

*Council*—Thomas Adams; James Andrews; Jonathan Barber; Rev. George Bathie, D.D.; Thomas Bedden; Benjamin Bensley; Clarke Burton; John Thomas Cooper; George Dudley; Thomas Fisher; Charles F. Forbes, M.D.; H. C. Hodge; Samuel Meadows; B. H. Smart; Peter Thomas; Richard Thompson; Thomas Tucker; Rev. T. M. Young, LL.B.

The Anniversary Oration was delivered by Dr. Gregory, and will shortly be published. It was very numerously attended, as was the dinner; and many excellent addresses were made by H. R. H. the Duke of Sussex, who was in the chair; by Lords Erskine, Henniker, &c.; Drs. Gregory, Mason, Collyer; Messrs. Coleridge, Pettigrew, &c. &c.—A volume of Transactions of the Society is now in the press, and will appear about the close of the present year.

---

*To the Editors of the London Medical and Physical Journal.*

GENTLEMEN,

I am requested to forward to you the following communication, under the impression that you will have the kindness to give it immediate publicity in the miscellaneous department of your Journal, and and in the hope, that, by so doing, the co-operation of our medical brethren in other parts of the kingdom will be excited in favour of an object of much importance to the general practitioner.

I am, &c.

CHRISTOPHER HEBB, Secretary to the Worcestershire Medical and Surgical Society.

*Worcester; June* 26, 1817.

At a general meeting of the members of the *Worcestershire Medical and Surgical Society*, it was resolved,—That the present system of removing paupers, on account of application in cases of illness to the overseers of the parish in which they happen to reside, to that parish to which they belong, often deprives the poor family of the means of gaining a living, and frequently induces them not to apply for a suspended order; while, if a medical man is called in, under such circumstances, to attend them, he has no legal means of obtaining any remuneration for his attendance.

Resolved,—That petitions be presented to both Houses of Parliament, praying that some regulation may be introduced in the Bill now pending relative to the poor-laws, for medical attendance upon the casual poor.

---

The *Royal Medical Society of Edinburgh* propose, as the subject of a Prize Essay for 1818, the following question:—"What Changes are produced on Atmospherical Air by the Action of the Skin of the Living Human Body." The members only are invited as candidates. The dissertations are to be written in English, French, or Latin, and to be delivered to the secretary, on or before the 1st of December. To each dissertation shall be prefixed a motto, and this motto is to be written on the outside of a sealed packet, containing the name and address of the author.

---

The Bulletin of the *Societé Medicale d'Emulation* contains a paper on the well-known custom among the African negroes, of eating a peculiar kind of earth. This, the writer imputes partly to an absolute deficiency of nourishment, but most to a choice in the nature of the earth, and in some respects to mental imbecility or aberration. The most important part of the paper is contained in the additional note of Messrs. Breschet and Cloquet.

"The singular disease, exhibited in the preceding Memoir, appears to be much more frequent in equatorial than northern regions. This, perhaps, depends on the want of real aliment being much less strongly felt under the torrid zone than in cold or temperate climates. Yet they think that the act of earth-eating is not decided by a peculiar taste, but by imperious necessity. Many nations, widely remote from each other, have the habit of allaying hunger by the ingestion of pure earth. M. de la Billardière asserts, that the inhabitants of New Caledonia possess only this kind of food during certain seasons of scarcity. When turtle-fishing is prevented by the inundation of the Orinoco, which lasts about three months, the the Ottomac nation is reduced to feed almost exclusively on a species of clay. Humboldt, by whom this fact is stated, asserts, that about a pound and a half of it, unmixed with crocodile fat, or any vegetable substance, is daily consumed by each individual. The only preparation consists in slightly frying, and afterwards moistening it.

"Somewhat analogous is observed by Golberry respecting the negroes of Los Idolos, islands at the mouth of the Senegal. They mix with their rice a mineral substance, which seems to serve as a substitute for butter.

"Brown says, that the crocodiles of South America swallow both small stones and pieces of wood, when the lakes which they commonly inhabit are dry, and they want food.

"Not far from Krasnoiarsk, on the river Jenissey, and in some mountains bordering on the Amour, is found a substance, called by the Russians, *kamennoïé maslo*, or rock butter. The elks and goats are singularly fond of it, and Patrin reports that it is employed by the hunters, as a bait wherewith to catch these animals.

"From all these facts may be drawn a somewhat curious general conclusion:—The earths, or stones, employed to distend the stomach, to allay hunger, or satisfy a depraved appetite, are almost invariably unctuous to the touch, fat, homogeneous, and contain much magnesia or alumine. Thus the author of the memoir has discovered that, at Martinique and Guadaloupe, the substance devoured by the negroes was an earth analogous to steatite, and formed by the decomposition of the porphyroid lava of the old volcanos of these islands.

"Vauquelin has analysed that of New Caledonia, and found it to consist of 0,37 of magnesia; 0,36 silex, and 0,17 oxide of iron. It is a green steatite, friable and tender.

"The earth of Los Idolos, is also a real steatite; but white, soft, and unctuous. Golberry eat of it without inconvenience or disgust.

"The rock butter forms stalactites in the cavities of the mountains alluded to. It is a mixture of argil, sulphate of alumine, sulphate of iron, and a small quantity of petroleum.

"One of these gentlemen asserts, that once, when pressed by hunger, he ate about five ounces of a lamellated talc, of a silver green colour, and very flexible, abounding in the mountains of the Tyrol. His appetite was satisfied without the slightest inconvenience.

"Most of the varieties of bolar and sigillated earths, which have been so long and frequently vaunted in the treatment of internal diseases, belong to the same class; but it is very probable, that their medical virtues depend upon the iron which enters into their composition."

In addition to the above, we would remark, that the substances consumed by chlorotic girls are usually the product of life. Chalk is generally supposed the effect of animal secretion, and pit-coal is often traced to a vegetable origin.

We

We some time ago hinted at an extraordinary optical phenomenon in Liverpool. We were then in hopes that a town, abounding with men of science, which can boast an Alanson, Curry, Roscoe, and a host of other living philosophers, which first instituted those docks the metropolis have at last imitated, and even led the way to a Lyceum, an Athenæum, and an Asylum for the Blind, would have at least discovered energy enough, either to detect imposture, or to ascertain a truly surprising fact; for it can now no longer be questioned, that Liverpool either contains a female who can see without eyes, or that she cannot be blinded. This is the only notice we shall at present take of this extraordinary subject.

---

*Extract from the Report of the House Committee of the Small-pox Hospital.*—"Your physician has reported, that the number of vaccinated subjects is greater, by 406, than during the first five corresponding months of the preceding year, and that the numbers inoculated are fewer; yet your committee have to lament, that small-pox has been more frequent, and its fatality greater, as appears by the reports of the weekly bills of mortality, as well as the numbers admitted into this house. Your committee have no reason, especially when they consider the report of the physician, to suppose, that this unhappy increase of small-pox has arisen from any distrust of vaccination, but rather from an inattention in the labouring class of society to their own healths and the lives of their children.

---

The following improved preparation of a very necessary medicine, we consider worth the notice of our readers. The suggestion is from Mr. Johnson, surgeon to the Lancaster Dispensary.

"The machine I use is similar to one made several years ago by Edmund Loyd and Co. 178, Strand; and does not differ essentially from any of those described in Count Rumford's 18th essay, and in the Repertory of Arts for April and May, 1813.

"Peruvian bark pounded and sifted through a wire sieve is placed in the strainer of this apparatus, and boiling water is poured upon it in successive portions. When about a quart of water has been thus passed through an ounce of cinchona, it forms a beautiful, clear solution of all that water can extract, and strongly exhibiting the sensible properties of cinchona.

"Dr. Duncan, jun. has long ago suggested that the precipitate which decoction of bark deposits on cooling may prove an useful and compendious medicine. This suggestion seems well founded, and deserves the trial. Those inclined to examine this matter may obtain a very abundant supply on cooling the strong infusion which first runs off.

"A little reflection on the chemical properties of cinchona, and a comparison of the infusion and decoction as usually prepared with the infusion just described, will convince your readers that the last preparation must afford an useful and efficient medicine. The saving effected in this way will be a small matter of consideration with medical practitioners as far as their interest only is concerned; but in dispensaries, infirmaries, and the public service, no item of expense is unimportant: and since on some occasions a scanty supply of cinchona has been felt as a national calamity, it becomes a duty to prevent a wasteful consumption of this valuable remedy. On this account, I mention, that in the Lancaster Public Dispensary this method is found to afford a better preparation than was formerly obtained from twice the quantity of cinchona.

"The stratum of bark should be of such a thickness that the water may neither pass through too slowly nor too rapidly. I have found a strainer of two inches, three inches, or four inches diameter, most suitable for half an ounce, one ounce, or two ounces, of cinchona."—*Annals of Phil.*

---

In our original department will be found a very interesting history of a living toad and newt incased in stone for a time beyond human calculation.—See p. 35.

---

*List of Patients admitted into all the Civil Hospitals of Paris, from the* 1*st of May, to the* 31*st of May,* 1817, *both inclusive.*

| | |
|---|---|
| Uncharacterised fevers | 35 |
| Ditto of various types | 248 |
| Ditto, bilious or gastric | 150 |
| Ditto, adynamic or putrid | 17 |
| Ditto, catarrhal | 4 |
| Phlegmasiæ, internal and external | 132 |
| Ophthalmia | 32 |
| Rheumatic pains | 22 |
| Diarrhœa and dysentery | 21 |
| Erysipelas | 7 |
| Phlegmasiæ of the organs of respiration | 172 |
| Consumptions | 47 |
| Apoplexy and recent paralysis | 20 |
| Dropsy and anasarca | 41 |
| Variolous | 9 |
| Metallic colics | 11 |
| Sporadic and chronic disorders and accidents | 227 |
| Itch | 107 |
| Total | 1302 |

---

*Reigning Maladies in Paris, from the* 1*st to the* 10*th of May.*

Summer has come briskly upon us; the north wind has ceased, but the drought continues with now and then a heavy shower. The accidents mentioned in our last are multiplied in the country. Inflammatory disorders are prevalent, principally of the mucous kind, or affecting the mucous membrane of the breast and belly.

*From the* 11*th to the* 20*th of May.*—A mild and seasonable temperature has replaced the weather complained of in our last. Fertile rains have reanimated our plains, and given joy and hope to all. The Centigrade thermometer is generally from 15 to 18 and

20 degrees in the middle of the day, and in the morning it does not descend to below 8 to 10.

Disorders continue to be inflammatory, and sometimes with extreme intensity. Peripneumonics have been so violent as to carry off their victims in twelve or fifteen hours. It is remarkable, that all the present disorders are accompanied with a tendency to affections of the brain, and we ought, therefore, to be more careful in the mode of treatment. Bleeding in the lower members, on the foot, or leeches on the arms, ought to be insisted on; these sanguine evacuations are rather to be performed as derivatives than depletives, and congestions in the brain have taken place from not attending to this. Able practitioners have added antispasmodics, as camphor, æther, valerian, &c. in cases of violent inflammation.

Women in child-bed have been frequently attacked with pleurisy, and even an inflammation of the meninges. Persons who are not well acquainted with this delicate point of science comprehend nothing of the progress of the disorder, but skilful physicians know that women in child-bed are much exposed to inflammation of the serous membranes, especially the peritoneum, which had been called puerperal fever before it was discovered by pathological anatomy that it was a local affection. They also know besides, that these disorders, always more or less modified by circumstances, do not always confine themselves to the peritoneum, but often extend to the pleura and the meninges, and even sometimes attack these latter membranes the first.

*From the* 21*st to the* 30*th of May.*—The temperature is mild, but unfortunately heavy rains fall every day, which have caused numerous gastric and bilious disorders. I have been singularly struck to find all the patients to whom I have been called, the same day, attacked with the same disorder.

Some of these are simply gastric obstructions, which have been removed by an emetic. In acute and nervous disorders, I have found these combined with old pulmonary catarrhs.

DE MONTEGRE, Editor of La Gazette de Santé.

[The English reader will be surprised at such feeble practice as the above, in diseases so violent as to carry off the patients in fifteen hours.]

---

### REPORT OF DISEASES.

THE weather has been extremely favourable to the health of invalids and the labouring class. Pulmonary complaints, in their chronic form, have almost disappeared; and the *febris pauperum* which threatened such ravages has been suddenly arrested. The employment furnished by the hay-harvest, brick-making, and all the labour attending building, repairing, and even the destruction of houses, has diffused a degree of activity which, with the almost constant appearance of an unclouded sun, restores to Augusta a prospect of her ancient industry, with every improvement of comfort and splendour. The magnificent structure of a bridge, superior rior to any thing England could boast, and by its level structure affording all the charms of novelty, and displaying at a single glance all its beauties, without concealing the distant hills, at the most favourable season for a landscape view, is the first prominent improvement which presents itself; and, though the public at large may not be aware how much such spectacles contribute to the general health, yet every medical man in full practice is sensible of the influence they produce on certain classes of patients, and certain descriptions of disease. This is evinced on a still larger scale, and with a force which defies all scepticism, in the health of an army or fleet, who, under the most favourable circumstances, are rarely kept in health without continual exercise of body and mind directed to some successful enterprize.

The ground to be cleared for the Post Office is supposed to be covered by 200 houses, almost all of them full of lodgers, and probably containing a population of more than 3,000 human beings. In the same neighbourhood, the governors of Christ's Hospital have dispossessed nearly 500 more. All these must seek an abode in more healthy spots; which is some relief to a mind reflecting on the temporary inconveniences to which many of the poorer inmates must be exposed. Add to this, fresh activity must be infused among the most abject by the erection of 700 houses in Spa-fields. To this spot, many, hitherto confined in the lower rooms of houses situated in dark alleys, will now repair, and, it is hoped, enjoy the comforts of a garden in addition to an airy frontage.

We have been led to these reflexions, not only by the influence such events have on the public health and the character of disease, but by the fallacious opinion some are apt to form of our increased number of inhabitants. That the population of London, generally so called, is greatly increased, cannot be questioned. But, whilst the single parish of Mary-le-bone, once an inconsiderable village, as its church proves, now possesses a population exceeding in number all Bristol and Clifton, with its environs; the interior part of the city of London is become an immense warehouse or bazaar, with its counting-houses. The apprehension, therefore, of Dr. Price, that England, like a ricketty child, had a head too large for its body, is unfounded. To pursue the allusion—its head is brought into shape, its shoulders spread, and, what is most to the purpose, its chest is expanded and supplied with purer air. We are doubtful whether London is not more free from acute diseases than most parts of the country.

We have, in vain, endeavoured to acquire further information concerning the fever in the Duke of Newcastle's family, and said to be sporadic in other parts of the country, particularly along the eastern coast. We have, however, reason to hope the accounts have been exaggerated, and, still more, that the fevers have not generally proved fatal. In London, the complaints are still inflammatory; but this is less universal among the higher classes, probably from their greater attention to their health, which induces them to seek relief before inflammation assumes a formidable aspect. However, ever, it is certain that an improved education has induced an improved mode of life, more consistent with steady health.

It is not a little remarkable, that the Reporter has seen three instances of epilepsy terminating fatally within the month, in constitutions exhausted by a complication of diseases. Two of these subjects were habitually intemperate; the third had, for nearly twelve months, shown symptoms of approaching dissolution. One of the former, a female, had been dropsical, and seemed to die from a sudden transfusion of water to the brain. The other, a male, passed bloody urine for more than a week before his death. Permission could not be obtained to examine either.

Most of the other complaints have been about the head, in the form of intense head-ach, or symptoms approaching apoplexy. All have been relieved by the lancet, or evacuation from the bowels.

---

### Mr. SALISBURY's *Botanical Excursions for June.*

Wednesday, June 4th.—Battersea Fields.

Geranium Pyrenaicum
Carex gracilis
——— riparia
——— acuta
——— vaginata
Ranunculus sceleratus
——————— auricomus
Symphytum tuberosum
Carduus palustris
———— heterophyllus
Tragopogon pratense
Poa fluitans
Lychnis Flos Cuculi
Alopecurus geniculatus
Veronica Scutelata
———— Beccabunga
———— Anagallis
Polygonum Bistorta
Cratægus Oxycantha
Fumaria officinalis
Equisetum limosum
Sisymbrium terrestre.

Wednesday, June 11th.—Same place.

Papaver Argemone
———— Rhœas
———— hybridum
———— Somniferum
Poa angustifolia
—— loliacea
—— cristata
Fistuca pinnata
Scandix Anthriscus
Narcissus Pseudo Narcissus
———— biflorus
Callitriche verna
Potamogetum lucens
———— crispum
Geranium parviflorum
———— rotundifolium
———— Robertianum
Scandex Pecten-Veneris
Bromus sterilis
Anthemis arvensis
Sinapis arvensis
———— alba
———— nigra
Myriophyllum verticillatum
Cochlearia officinalis
Anagallis arvensis
Urtica urens
——— dioica
Trifolium procumbens
Cerastium viscosum
Trifolium pratense
———— repens
Carex depauperata
——— vulpina
Galium palustre.

Wednesday, June 18th.—Combe-hurst, near Croydon.

Poa nemoralis

——— pratensis

——— trivialis

Lysimachia Nemorum

———— Nummularia

Ajuga reptans

Euphorbia

Euphorbia amygdaloides

Mercurialis perennis

Geranium dissectum

Lychnis dioica flore alba

Sherrardia arvensis

Galium pusillum

Asperula odorata

Lotus corniculatus

Orchis bifolia

——— maculata

——— monorchis

——— mascula

Ophrys ovata

——— Anthropophora

——— apifera

Polypodium fontanum

——— vulgaris

Geranium Columbinum

Carduus nutans

Festuca duriuscula

Festuca myuros

Œnothera biennis

Carduus marianus

Hieracium pilosella

Veronica officinalis

Tormentilla recta

Hippocrepis cernosa

Plantago Coronopus

Polygonum Aviculare

Polemonium cæruleum

Alopecurus agrestis

Litherpermum arvense

Aira flexuosa

Bromus muralis

Carex stellulata

Aspedium Filix Mas

——— fœmina

Stellera uliginosa

Carex remota

Bunium Bulbocastanum

Rhamnus Frangula

Scrophularia nodosa

Euonymus europæus

Poa cristata

— subsærulea

— compressa.

Saturday, June 21st.—Battersea Fields.

Festuca glauca
Dactylus glomerata
Cynoglossum officinale
Senecio Jacobæa
Chrysanthemum Leucanthemum
Medicago lupulina
——— sativa
——— varia
Reseda lutea
——— Luteola
Carduus lanceolatus
Solanum Dulcamara
Tamus communis
Bryonia alba
Cucubulus Behen
Geum urbanum
Carex muricata
Vicia sativa
Lathyrus pratensis
Papaver dubium
Saxifraga umbrosa
Vicia sepium
——— craccea
Leontodon hispidum
Hydrochæris Morsus-Ranæ
Myosotis Scorpioides.

Wednesday, June 25th.—Regent's Park and the neighbourhood of Copenhagen-house.

Avena flavescens
——— fatua
Alopecurus crinitus
Plantago media
Alisma Plantago
Poa retroflexa
— aquatica
Crepis biennis
Malva sylvestris
Sonchus oleraceus
Sonchus arvensis
Antirrhinum majus
——— Orontium
——— minus
——— Cymbalaria
Conium maculatum
Daucus Carota
Sambucus Ebulus
——— nigra.

The days of the Excursions in July will be made known by application at the Botanic Garden at Sloane-street, as usual.

The crop of grass, which is now cutting, is abundant, and some hay is already made and carried. The spring-sown corn, owing to the late dry weather, is very backward, and appears to be come up rather rather *unevenly*, which is unfavourable for such crops. Barley in particular, when it appears above ground at different times will often preserve a difference in growth till harvest, and is then the cause of the grain being of different degrees of maturity when reaped. There appears to be only a very moderate crop of apples, and but little prospect of an abundant crop of other kinds of standard fruit near London.

*June*, 1817.

---

### A METEOROLOGICAL JOURNAL,

By Messrs. WILLIAM HARRIS and Co. 50, Holborn, London.

*From the 20th May to the 19th June, inclusive.*

| Day of Month. | Rain gauge | Therm. | | | Barom. | | De Luc's Hygrom. Dry | | Damp | | Wind. | | Atmospheric Variation. | | |
|---|---|---|---|---|---|---|---|---|---|---|---|---|---|---|---|
| May | | | | | | | | | | | | | | | |
| 20 | | 45 | 49 | 42 | $29^5$ | $29^5$ | | | 2 | 2 | NE | NE | Rain | | |
| 21 | ,29 | 47 | 51 | 40 | $29^4$ | $29^6$ | | | 2 | 5 | N | SW | Rain | | Clo. |
| 22 | ,20 | 43 | 47 | 40 | $29^5$ | $29^5$ | | | 3 | 2 | SW | SW | Fine | Rain | Clo. |
| 23 | ,12 | 42 | 52 | 43 | $29^5$ | $29^5$ | | | 4 | 4 | SW | SSW | Clo. | Fine | |
| 24 | | 49 | 51 | 42 | $29^5$ | $29^5$ | | | 3 | 3 | SW | S | Fine | Rain | Fine |
| 25 | ,18 | 44 | 47 | 41 | $29^2$ | $29^2$ | | | 4 | 4 | SE | NE | Rain | | Fine |
| 26 | ,23 | 45 | 51 | 47 | $29^2$ | $29^4$ | | | 4 | 4 | SE | SE | Clo. | | |
| 27 | ,10 | 51 | 59 | 46 | $29^5$ | $29^5$ | | | 4 | 4 | SE | S | Fine | Rain | Cl. |
| 28 | ,13 | 51 | 60 | 47 | $29^6$ | $29^7$ | | | 5 | 5 | S | NNW | Clo. | Rain | Clo. |
| 29 | ,09 | 48 | 54 | 45 | $29^5$ | $29^7$ | | | 5 | 4 | NE | N | Rain | | Fine |
| 30 | ,24 | 48 | 53 | 41 | $29^7$ | $29^9$ | | | 3 | 2 | NNE | N | Clo. | | |
| 31 | ,17 | 16 | 59 | 44 | $29^7$ | $29^8$ | | | 1 | 2 | NW | W | Fine | | |
| June | | | | | | | | | | | | | | | |
| 1 | | 52 | 56 | 44 | $29^8$ | $29^8$ | | | 2 | 2 | W | SW | Fine | Clo. | Rain |
| 2 | ,12 | 51 | 59 | 48 | $29^8$ | $29^7$ | | | 3 | 2 | W | SW | Fine | Clo. | |
| 3 | | 54 | 61 | 52 | $29^7$ | $29^6$ | | | 1 | 3 | SSW | S | Fine | | Rain |
| 4 | ,10 | 56 | 61 | 49 | $29^5$ | $29^9$ | | | 4 | 4 | S | SW | Rain | Clo. | Fine |
| 5 | ,08 | 54 | 62 | 52 | 30 | $30^4$ | | | 4 | 3 | SW | SSW | Fine | | |
| 6 | | 56 | 63 | 59 | 30 | $29^9$ | | | 2 | 3 | SW | SSW | Fine | | Clo. |
| 7 | | 66 | 70 | 54 | $29^9$ | $29^7$ | | | 3 | 3 | SW | SW | Fine | | Clo. |
| 8 | | 59 | 68 | 53 | $29^9$ | 30 | | | 3 | 2 | SW | SW | Rain | Clo. | Fine |
| 9 | ,15 | 57 | 69 | 55 | 30 | $30^1$ | 0 | | 0 | 1 | SW | SW | Fine | | Clo. |
| 10 | | 56 | 68 | 56 | 30 | 30 | | | 2 | 4 | W | SW | Rain | | Clo. |
| 11 | ,17 | 59 | 65 | 52 | $29^9$ | $29^8$ | | | 5 | 4 | S | S | Fine | | |
| 12 | | 55 | 61 | 53 | $29^7$ | $29^7$ | | | 3 | 5 | SE | SE | Rain | Clo. | |
| 13 | ,10 | 55 | 59 | 54 | $29^6$ | $29^3$ | | | 5 | 5 | E | SW | Rain | | Clo. |
| 14 | .14 | 57 | 59 | 54 | $29^4$ | $29^9$ | | | 4 | 3 | SSW | WSW | Fine | Rain | Clo. |
| 15 | ,07 | 59 | 61 | 47 | $30^1$ | $30^2$ | 0 | 1 | | | W | N | Fine | | |
| 16 | | 55 | 61 | 49 | $30^2$ | $30^2$ | 0 | 0 | 0 | 0 | N | SSE | Fog | Fine | |
| 17 | | 57 | 69 | 59 | 30 | $29^8$ | | | 1 | 3 | E | NE | Fine | | |
| 18 | | 63 | 77 | 63 | $29^8$ | $29^7$ | | | 3 | 2 | NNE | ENE | Fine | | |
| 19 | ,02 | 66 | 77 | 67 | $29^7$ | $29^8$ | | | 1 | 1 | SE | S | Fine | | |

The quantity of rain fallen in the month of May is 2 inches and 78-100ths.

